# Abnormally High Levels of Virus-Infected IFN-γ^+^CCR4^+^CD4^+^CD25^+^ T Cells in a Retrovirus-Associated Neuroinflammatory Disorder

**DOI:** 10.1371/journal.pone.0006517

**Published:** 2009-08-05

**Authors:** Yoshihisa Yamano, Natsumi Araya, Tomoo Sato, Atae Utsunomiya, Kazuko Azakami, Daisuke Hasegawa, Toshihiko Izumi, Hidetoshi Fujita, Satoko Aratani, Naoko Yagishita, Ryoji Fujii, Kusuki Nishioka, Steven Jacobson, Toshihiro Nakajima

**Affiliations:** 1 Department of Molecular Medical Science, Institute of Medical Science, St. Marianna University School of Medicine, Kawasaki, Japan; 2 Department of Hematology, Imamura Bun-in Hospital, Kagoshima, Japan; 3 Viral Immunology Section, Neuroimmunology Branch, National Institute of Neurological Disorders and Stroke, National Institutes of Health, Bethesda, Maryland, United States of America; 4 Department of Genome Science, Institute of Medical Science, St. Marianna University School of Medicine, Kawasaki, Japan; BMSI-A*STAR, Singapore

## Abstract

**Background:**

Human T-lymphotropic virus type 1 (HTLV-1) is a human retrovirus associated with both HTLV-1-associated myelopathy/tropical spastic paraparesis (HAM/TSP), which is a chronic neuroinflammatory disease, and adult T-cell leukemia (ATL). The pathogenesis of HAM/TSP is known to be as follows: HTLV-1-infected T cells trigger a hyperimmune response leading to neuroinflammation. However, the HTLV-1-infected T cell subset that plays a major role in the accelerated immune response has not yet been identified.

**Principal Findings:**

Here, we demonstrate that CD4^+^CD25^+^CCR4^+^ T cells are the predominant viral reservoir, and their levels are increased in HAM/TSP patients. While CCR4 is known to be selectively expressed on T helper type 2 (Th2), Th17, and regulatory T (Treg) cells in healthy individuals, we demonstrate that IFN-γ production is extraordinarily increased and IL-4, IL-10, IL-17, and Foxp3 expression is decreased in the CD4^+^CD25^+^CCR4^+^ T cells of HAM/TSP patients as compared to those in healthy individuals, and the alteration in function is specific to this cell subtype. Notably, the frequency of IFN-γ-producing CD4^+^CD25^+^CCR4^+^Foxp3^−^ T cells is dramatically increased in HAM/TSP patients, and this was found to be correlated with disease activity and severity.

**Conclusions:**

We have defined a unique T cell subset—IFN-γ^+^CCR4^+^CD4^+^CD25^+^ T cells—that is abnormally increased and functionally altered in this retrovirus-associated inflammatory disorder of the central nervous system.

## Introduction

Human T-lymphotropic virus type 1 (HTLV-1) is a human retrovirus associated with chronic and persistent infection of human T cells. While the majority of infected individuals remain healthy lifelong asymptomatic carriers, approximately 3–5% develop aggressive mature T-cell malignancy termed adult T-cell leukemia (ATL) and another 0.25–3% develop a chronic neuroinflammatory disease termed HTLV-1-associated myelopathy/tropical spastic paraparesis (HAM/TSP) [1]–[4]. Furthermore, in some HAM/TSP patients, other autoimmune diseases characterized by multiorgan lymphocytic infiltrates, including uveitis, Sjögren syndrome, arthritis, polymyositis, and alveolitis have been reported [5], [6]. HAM/TSP patients have high frequency of HTLV-1-infected T cells, heightened viral gene expression (in particular, the expression of the viral transactivating gene *tax*), and virus-specific immune responses, including increased production of proinflammatory cytokines such as IL-6, IL-12, and IFN-γ [7]–[9]. Neuropathological findings have demonstrated focal infiltrates of HTLV-1-infected CD4^+^ T cells, CD8^+^ T cells, and macrophages in the central nervous system (CNS) [10]. It has been suggested that together with viral gene expression and cellular signaling mechanisms, HTLV-1-infected T cells trigger a strong virus-specific T cell response, leading to CNS inflammation and autologous tissue damage. We hypothesized [11], [12] that these “pathogenic” HTLV-1-infected T cells may trigger chronic hyperimmune responsiveness in HAM/TSP patients, and thus they might be an important target for clinical therapeutic interventional strategies.

HTLV-1 has been reported to infect a number of cell types in vitro and in vivo [13]–[16]. In our previous study, we used molecular and immunological techniques to demonstrate that CD4^+^CD25^+^ T cells are the predominant viral reservoir in the peripheral blood [12]. In healthy individuals, subsets of CD4^+^CD25^+^ T cells, termed regulatory T cells or Treg cells, has been demonstrated to be hyporesponsive to antigenic stimulation and is reported to suppress the proliferation and cytokine production of CD4^+^CD25^−^ and CD8^+^ effector T cells in vitro [17]. Although Treg cells are phenotypically similar to activated T cells, they can be identified *ex vivo* by the intracellular expression of the transcriptional regulator Foxp3 [18], which is critical for the development and function of Treg cells in both mice and humans [17]. Previous studies have observed significant reductions in Foxp3 expression and/or Treg function in several human autoimmune diseases, including multiple sclerosis (MS), myasthenia gravis, and type I diabetes [17], [19], suggesting that defects in Foxp3 expression and/or Treg function may precipitate the loss of immunological tolerance. In our recent study, we reported that CD4^+^CD25^+^ T cells from HAM/TSP patients exhibited reduced Foxp3 expression levels compared to healthy individuals and that this reduction was associated with a loss of suppressor function [20]. Furthermore, overexpression of a single HTLV-1 gene (*tax*) was capable of reducing Foxp3 expression and dampening the suppressive function of healthy donor CD4^+^CD25^+^ T cells [20]. These observations suggest that persistent T cell activation in HAM/TSP patients triggered by viral antigens, e.g., HTLV-1 tax, that are associated with Foxp3 downregulation may result in decline of the CD4^+^CD25^+^Foxp3^+^ Treg cell population and accumulation of CD4^+^CD25^+^Foxp3^−^ T cells that lack suppressive function but are capable of exacerbating the disease process. Therefore, we hypothesize that in HAM/TSP patients, the Foxp3^−^ HTLV-1-infected CD4^+^CD25^+^ T cells may contain an immunopathogenic T cell subset that can accelerate the immune response. Thus far, however, the role of Foxp3-negative CD4^+^CD25^+^ T cells has not been addressed in the pathogenesis of HAM/TSP.

To detect these HAM/TSP “pathogenic T cell” subsets from the pool of HTLV-1-infected T cells, it was important to define additional immunological markers of HTLV-1 infection in HAM/TSP patients. Although ATL cells have also been shown to have HTLV-1-infected CD4^+^ cells with high expression of CD25^+^ [21], a recent study demonstrated that ATL cells expressed the chemokine receptor CCR4, which is known to be selectively expressed on T helper type 2 (Th2) cells and Treg cells subsets [22]. More recently, it has been observed that CCR4 is expressed on Th17 cells [23]. Furthermore, it has been reported that HTLV-1 preferentially transmits to CCR4^+^CD4^+^ T cells through the CCR4 ligand (CC chemokine ligand (CCL) 22), which is induced by *tax* and consequently expressed on the surface of HTLV-1-infected T cells [24]. However, it has been demonstrated that in HAM/TSP patients, HTLV-1-infected CD4^+^CD25^+^ T cells produce the Th1 cytokine IFN-γ and contain fewer Foxp3^+^ Treg cells [20]. Therefore, it would be of interest to determine the percentage of CCR4-expressing cells in the HAM/TSP CD4^+^CD25^+^ subset and whether CCR4 can be used as a marker of HTLV-1-infected T cells in HAM/TSP.

In the present study, we demonstrated that CD4^+^CD25^+^CCR4^+^ T cells are the main reservoir for HTLV-1 in HAM/TSP patients. While CD4^+^CD25^+^CCR4^+^ T cells in ATL showed high Foxp3 expression (Treg), this subset showed low Foxp3 expression in HAM/TSP, and this Foxp3^−^ T cell subset actively proliferated in HAM/TSP patients. Notably, the CD4^+^CD25^+^CCR4^+^ T cells in HAM/TSP patients were producing extraordinarily high levels of IFN-γ, while this T cell subset produced low levels of IFN-γ in healthy donors. Furthermore, the expression levels of IL-2, IL-4, IL-10, and IL-17 in the CD4^+^CD25^+^CCR4^+^ T cells in HAM/TSP patients were low compared to the corresponding levels produced by this T cell subset in healthy individuals. Further, it is noteworthy that these functional alterations were specific to the CD4^+^CD25^+^CCR4^+^ T cell subtype (HTLV-1-infected T cells). Moreover, the frequency of these IFN-γ-producing CD4^+^CD25^+^CCR4^+^Foxp3^−^ T cells was increased and found to be correlated with disease severity in HAM/TSP patients. Thus, our study is the first to demonstrate that abnormal IFN-γ-producing CCR4^+^CD4^+^CD25^+^ T cells, which are rare in healthy individuals, are increased in HTLV-1-infected T cells in HAM/TSP patients.

## Results

### CD4^+^CD25^+^CCR4^+^ T cells are the main reservoir of HTLV-1 and are present in high numbers in HAM/TSP patients

**Figure 1:**
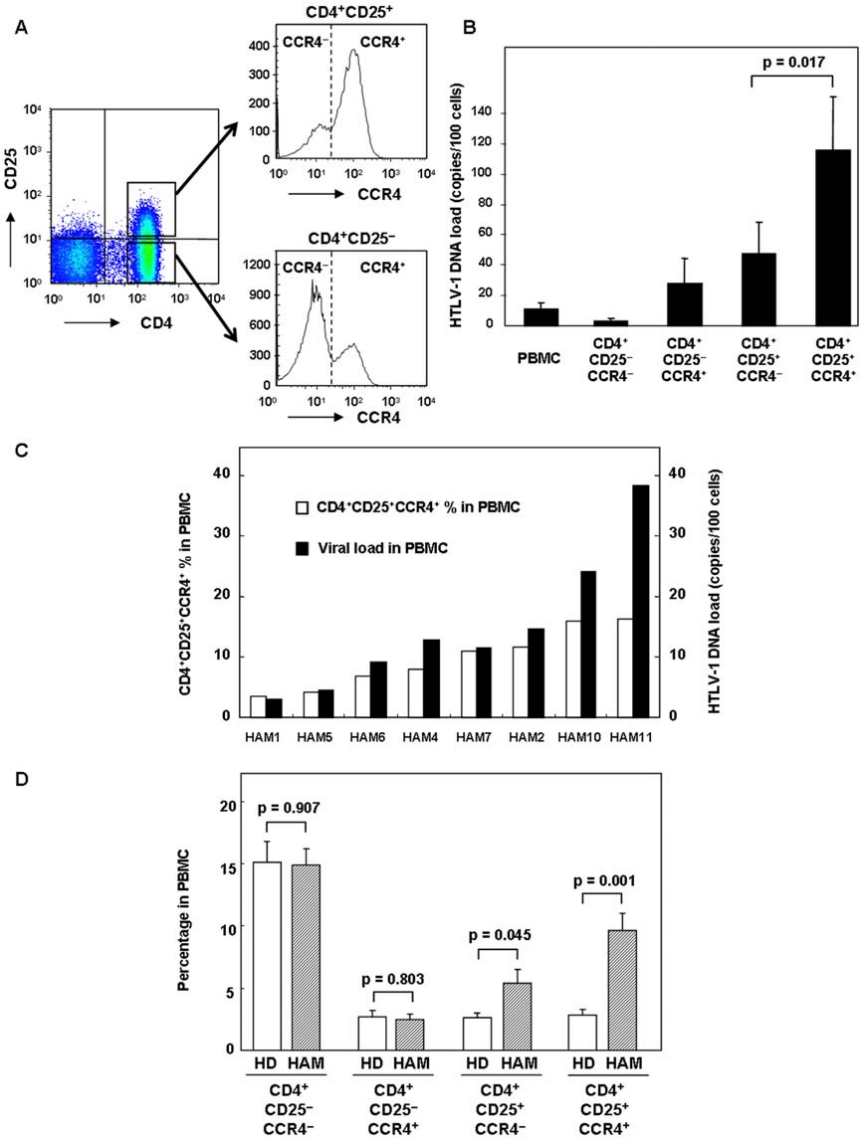
CD4^+^CD25^+^CCR4^+^ T cells are the main reservoir for HTLV-1 and are present in increased levels in HAM/TSP patients. A. Representative plot of FACS sorting. CD4^+^CD25^−^CCR4^−^, CD4^+^CD25^−^CCR4^+^, CD4^+^CD25^+^CCR4^−^, and CD4^+^CD25^+^CCR4^+^ T cells were separated by FACS. The purity of each cell population was approximately 99% (Figure S1). B. HTLV-1 proviral DNA load of PBMCs and CD4^+^CD25^−^CCR4^−^, CD4^+^CD25^−^CCR4^+^, CD4^+^CD25^+^CCR4^−^, and CD4^+^CD25^+^CCR4^+^ T cells were assessed using quantitative PCR in 8 HAM/TSP patients. Data are presented as mean±SE. The HTLV-1 proviral load was significantly higher in the CD4^+^CD25^+^CCR4^+^ T cells than in the CD4^+^CD25^+^CCR4^−^ T cells (p = 0.017 by the Wilcoxon signed-rank test). C. HTLV-1 proviral DNA load in PBMCs and the percentages of CD4^+^CD25^+^CCR4^+^ T cells in the PBMCs of 8 HAM/TSP patients. D. The frequency of CD4^+^CD25^−^CCR4^−^, CD4^+^CD25^−^CCR4^+^, CD4^+^CD25^+^CCR4^−^, and CD4^+^CD25^+^CCR4^+^ T cells in HDs and HAM/TSP patients. The frequency of CD4^+^CD25^−^CCR4^−^, CD4^+^CD25^−^CCR4^+^, CD4^+^CD25^+^CCR4^−^, and CD4^+^CD25^+^CCR4^+^ T cells in 8 HDs and 11 HAM/TSP patients was analyzed by FACS and represented as the frequency of each cell population. Data are presented as mean±SE. The P value was calculated by unpaired Student's *t* test.

To determine whether CCR4 can be used as a cell surface marker to identify HTLV-1-infected T cells in HAM/TSP patients as reported in ATL cells [22], we separated peripheral blood mononuclear cells (PBMCs) from HAM/TSP patients into the following T cell subtypes by FACS sorting: CD4^+^CD25^−^CCR4^−^, CD4^+^CD25^−^CCR4^+^, CD4^+^CD25^+^CCR4^−^, and CD4^+^CD25^+^CCR4^+^ (Figure 1A). The purity of each separated cell subtype was approximately 99% (Figure S1). The HTLV-1 proviral DNA load was then determined within each population by real-time quantitative PCR. As shown in Figure 1B (means of 8 HAM/TSP patients), the HTLV-1 proviral DNA load was higher in CD4^+^CD25^+^ T cells than in CD4^+^CD25^−^ T cells; this result is consistent with those of previous studies [12]. Moreover, when CCR4 was used to segregate these T cell subsets, increased HTLV-1 provirus levels were also observed in CD4^+^CCR4^+^ cells, including CD25^−^ cells. Importantly, the CD4^+^CD25^+^CCR4^+^ T cell subset was infected with HTLV-1 at significantly higher frequencies than for the CD4^+^CD25^+^CCR4^−^ T cell subset (p = 0.017). If the HTLV-1 provirus integrates at the rate of 1 copy per cell [14], [25], the proviral DNA load (115.65±35.28 copies/100 cells) in CD4^+^CD25^+^CCR4^+^ T cells suggests that the majority (if not all cells) of this population is infected with HTLV-1.

These results directly demonstrated that CD4^+^CD25^+^CCR4^+^ T cells are the main virus reservoir in HAM/TSP patients; therefore, we aimed to determine the percentage of this T cell subset in the PBMCs to the total HTLV-1 proviral DNA in each HAM/TSP patient. As shown in Figure 1C, the amount of HTLV-1 proviral DNA (which is the percent of HTLV-1-infected cells in PBMCs, provided the provirus integrates at the rate of 1 copy per cell) and the percentage of CD4^+^CD25^+^CCR4^+^ T cells were comparable in the HAM/TSP patients with low to medium HTLV-1 viral loads (HAM 1, HAM 5, HAM 6, HAM 4, HAM 7, and HAM 2). In the HAM/TSP patients with higher virus loads (HAM 10 and 11), the number of virus-infected cells (virus load) was demonstrated to be higher than the percentage of CD4^+^CD25^+^CCR4^+^ T cells, suggesting that other cell populations may also be infected in these patients.

As shown in the histogram plot in Figure 1A, CD4^+^CD25^+^CCR4^+^ cells exhibited a high peak while CD4^+^CD25^+^CCR4^−^ and CD4^+^CD25^−^CCR4^+^ cells exhibited low peaks in a representative HAM/TSP patient. To analyze whether this increased frequency is specific to the cell subtype, we analyzed the frequency of each cell population in 8 healthy donors (HD) and 11 HAM/TSP patients (Figure 1D). CD4^+^CD25^+^CCR4^+^ cells were significantly increased (p = 0.001) and CD4^+^CD25^+^CCR4^−^ cells were slightly increased (p = 0.045) in the HAM/TSP patients compared to the respective values in the HDs. Furthermore, the CD4^+^CD25^−^CCR4^+/−^ cell populations were almost equivalent in the HAM/TSP patients and HDs. Overall, the CD4^+^CD25^+^CCR4^+^ T cells were preferentially infected by HTLV-1, and they were found in higher numbers in HAM/TSP patients.

### Foxp3-negative CD4^+^CD25^+^CCR4^+^ T cells are higher in HAM/TSP patients than in healthy donors

**Figure 2:**
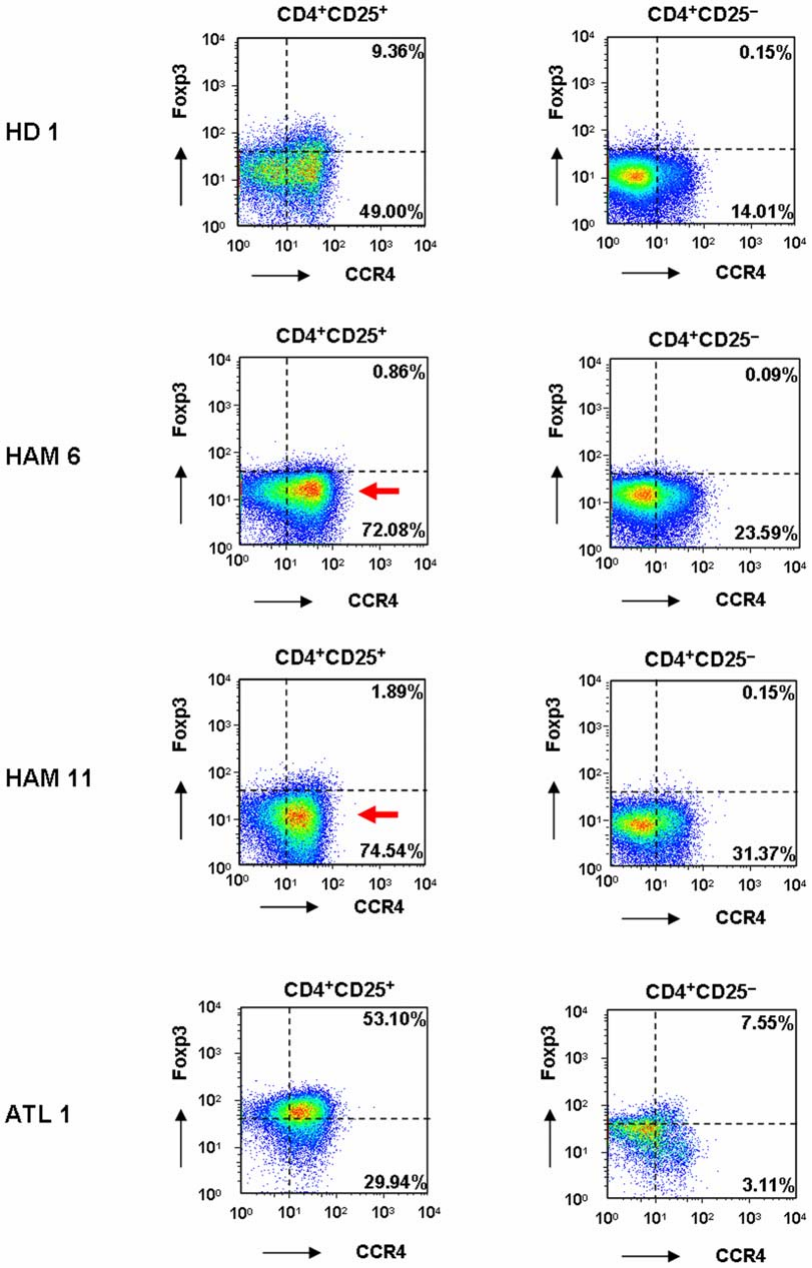
Different expression levels of FoxP3 in CD4^+^CD25^+^CCR4^+^ T cells between HD, HAM/TSP, and ATL. PBMCs from 1 healthy donor (HD 1), 2 HAM/TSP patients (HAM 6, HAM 11), and 1 ATL patient (ATL 1) were stained for CD4, CD25, CCR4, and intracellular Foxp3. CD4^+^CD25^+^ or CD4^+^CD25^−^ cells were gated, and the expression levels of CCR4 and Foxp3 were plotted. The percentages of CCR4^+^Foxp3^+^ cells and CCR4^+^Foxp3^−^ cells in CD4^+^CD25^+^ or CD4^+^CD25^−^ cells are indicated in each panel. The arrow indicates the increased number of cells with low Foxp3 expression levels in HAM/TSP CD4^+^CD25^+^CCR4^+^ T cells.

Although CCR4 is known to be selectively expressed on Th2, Th17, and Treg cells [23], [26] in healthy individuals, the expression levels of Foxp3, a hallmark of Treg cells [17], [18], has been reported to be reduced in HAM/TSP patients [20], [27]–[29]. Therefore, it is interesting to compare the Foxp3 expression levels in CD4^+^CD25^+^CCR4^+^ T cells between HAM/TSP patients, ATL patients, and uninfected healthy donors (HDs). As reported previously [30], only the CCR4^+^ subset of CD4^+^CD25^+^ cells expressed Foxp3^+^, whereas CD4^+^CD25^−^ cells in HD did not express Foxp3 (upper panels, Figure 2). Foxp3 expression in CD4^+^CD25^+^CCR4^+^ T cells was reduced in HAM/TSP patients and increased in ATL patients (Figure 2); this finding is in line with the results of previous studies regarding CD4^+^CD25^+^ T cells from HAM/TSP and ATL patients [27]–[29], [31]. The slightly elevated Foxp3 expression in the CD4^+^CD25^−^ population of ATL patients (lower right panel, Figure 2) may reflect the presence of leukemia cells. However, a more detailed analysis of these FACS plots demonstrated an increased number of Foxp3^−^ cells in the CD4^+^CD25^+^CCR4^+^ subset in HAM/TSP patients (arrow, Figure 2). This observation suggested that the number of CD4^+^CD25^+^CCR4^+^Foxp3^−^ T cells may be increased in HAM/TSP patients.

**Figure 3:**
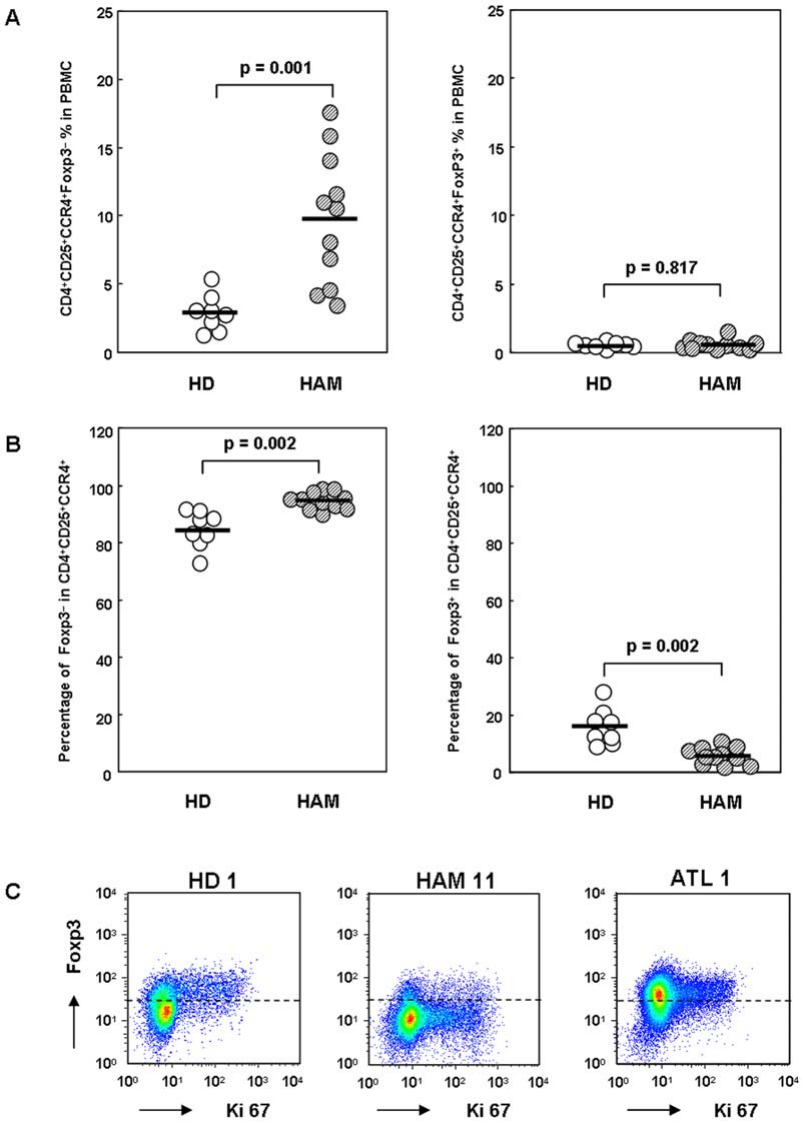
The Foxp3-negative CD4^+^CD25^+^CCR4^+^ T cells are higher in HAM/TSP patients than in healthy donors. A. Percentage of the CD4^+^CD25^+^CCR4^+^Foxp3^−^ or CD4^+^CD25^+^CCR4^+^Foxp3^+^ T cells in the total PBMCs in HD and HAM/TSP patients. PBMCs from 8 HD and 11 HAM/TSP patients were stained for CD4, CD25, CCR4, and intracellular Foxp3. The percentage of CD4^+^CD25^+^CCR4^+^Foxp3^−^ (left panel) or CD4^+^CD25^+^CCR4^+^Foxp3^+^ (right panel) T cells to the total PBMCs was calculated by FACS analysis. The horizontal bar indicates the mean value of each group. An unpaired Student's *t* test was used for statistical analysis. B. Percentage of the Foxp3^−^ or Foxp3^+^ population in CD4^+^CD25^+^CCR4^+^ T cells. PBMCs from 8 HDs and 11 HAM/TSP patients were stained for CD4, CD25, CCR4, and intracellular Foxp3. Then, the percentage of the Foxp3^−^ (left panel) or Foxp3^+^ (right panel) population in CD4^+^CD25^+^CCR4^+^ T cells was calculated. The horizontal bar indicates the mean value of each group. The percentage of the Foxp3^−^ population in CD4^+^CD25^+^CCR4^+^ T cells was significantly higher in the HAM/TSP patients than in the HDs (p = 0.002, unpaired Student's *t* test), and the percentage of the Foxp3^+^ population in the CD4^+^CD25^+^CCR4^+^ T cells was significantly lower in the HAM/TSP patients than in the HDs (p = 0.002, unpaired Student's *t* test). C. Foxp3 and Ki67 expression in CD4^+^CD25^+^CCR4^+^ T cells from HD 1, HAM 11, and ATL 1. CD4^+^ T cells were negatively separated by magnetic beads and stained for CCR4, CD25, and intracellular Foxp3 and Ki67. CD25^+^CCR4^+^ cells were gated, and the expression levels of Ki67 and Foxp3 were plotted.

Analysis of these T cell subsets based on Foxp3 expression in CD4^+^CD25^+^CCR4^+^ cells demonstrated a significant elevation in the frequency of CD4^+^CD25^+^CCR4^+^Foxp3^−^ T cells in the total PBMCs in HAM/TSP patients (n = 11) as compared to that in HDs (n = 8) (left panel, Figure 3A; p = 0.001). The percentage of Foxp3^−^ cells in the CD4^+^CD25^+^CCR4^+^ subset was also higher in HAM/TSP patients than in HDs (left panel, Figure 3B; p = 0.002). Further, the percentage of Foxp3^+^ cells in this subset was decreased in HAM/TSP patients than in HDs (right panel, Figure 3B; p = 0.002), and the frequency of Foxp3^+^ cells in this subset in the total PBMCs was equivalent in HAM/TSP patients and HDs (right panel, Figure 3A; p = 0.817).

To determine whether increased CD4^+^CD25^+^CCR4^+^Foxp3^−^ T cells in HAM/TSP patients were proliferating in vivo, we examined the expression levels of Foxp3 and Ki67 in CD4^+^CD25^+^CCR4^+^ T cells. Ki67 is a marker of proliferating cells. As shown in Figure 3C, Ki67-positive cells were predominantly confined to the CD4^+^CD25^+^CCR4^+^Foxp3^+^ T cells (defined as Treg cells) in HDs and ATL patients. On the other hand, CD4^+^CD25^+^CCR4^+^Foxp3^−^ T cells were preferentially positive for Ki67 in HAM/TSP patients, suggesting that the CD4^+^CD25^+^CCR4^+^Foxp3^−^ T cells were proliferating *in vivo*. Collectively, these results indicate that Foxp3 expression in HTLV-1-infected CD4^+^CD25^+^CCR4^+^ T cells is suppressed in HAM/TSP patients; this results in the accumulation and proliferation of the CD4^+^CD25^+^CCR4^+^Foxp3^−^ T cell subset.

### CD4^+^CD25^+^CCR4^+^Foxp3^−^ T cells produce multiple proinflammatory cytokines and overproduce IFN-γ in HAM/TSP

**Figure 4:**
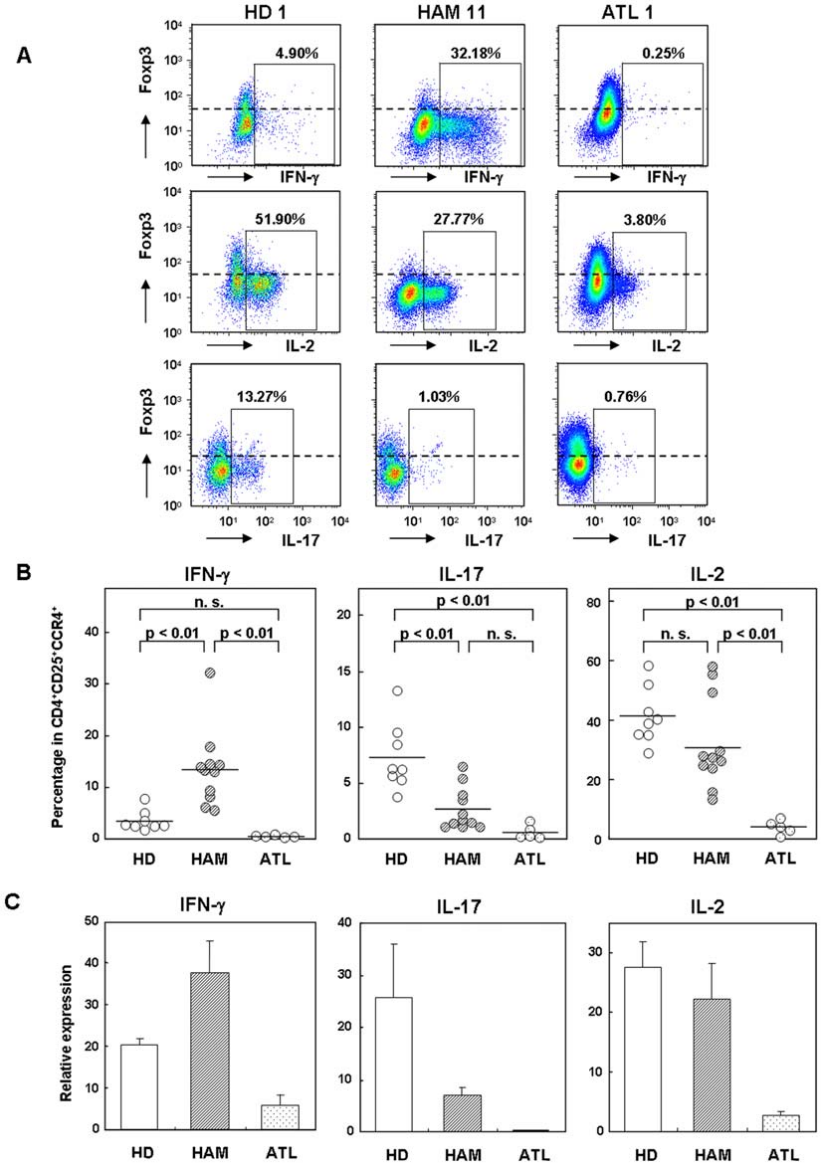
CD4^+^CD25^+^CCR4^+^Foxp3^−^ T cells produce multiple proinflammatory cytokines and overproduce IFN-γ in HAM/TSP. A. Representative proinflammatory cytokine expression in CD4^+^CD25^+^CCR4^+^ T cells from HDs, HAM/TSP patients, and ATL patients. CD4^+^ T cells were negatively separated by magnetic beads, and stimulated for 5 h with PMA and ionomycin in the presence of monensin. The cells were then stained for CCR4, CD25, intracellular Foxp3, and IFN-γ or IL-2 or IL-17A. The CD25^+^CCR4^+^ cells were gated, and the expression levels of each cytokine and Foxp3 were plotted. Numbers adjacent to the outlined area indicate the percentage of cytokine-producing cells in the CD4^+^CD25^+^CCR4^+^ T cell subset. B. The percentage of proinflammatory cytokine (IFN-γ, IL-17A, and IL-2) expression in CD4^+^CD25^+^CCR4^+^ T cells from 8 HDs, 11 HAM/TSP patients, and 5 ATL patients. The horizontal bar indicates the mean value of each group. Scheffe's test (nonparametric) was used for statistical analysis. n.s. not significant. C. Real-time RT-PCR of IFN-γ, IL-17A, and IL-2 mRNA in CD4^+^CD25^+^CCR4^+^ T cells separated by FACS sorting from 3 HDs, 4 HAM/TSP patients, and 3 ATL patients. Data are presented as mean±SE.

CCR4-expressing CD4^+^ T cells include Th2, Th17, and Treg cells, and they do not tend to produce Th1 cytokines such as IFN-γ in healthy individuals [26]. However, it has been reported that HTLV-1-infected CD4^+^ T cells in HAM/TSP patients can produce IFN-γ [20], [32]. Therefore, we investigated the intracellular proinflammatory cytokine expression in the CD4^+^CD25^+^CCR4^+^ T cells in 8 HDs, 11 HAM/TSP patients, and 5 ATL patients after phorbol 12-myristate 13-acetate (PMA) and ionomycin stimulation and compared the expression levels with those of Foxp3 (Figure 4A, B). Interestingly, the expression of IFN-γ, IL-2, and IL-17 was observed only in the Foxp3-negative population of CD4^+^CD25^+^CCR4^+^ T cells but not in the Foxp3-positive population (Treg cells) of CD4^+^CD25^+^CCR4^+^ T cells in HDs (Figure 4A). In HAM/TSP patients, IFN-γ production was demonstrated in the Foxp3-negative population of the CD4^+^CD25^+^CCR4^+^ T cells and was found to be significantly increased compared to that in HDs, while IL-2 and IL-17 production was decreased in HAM/TSP patients than in HDs. In ATL patients, although PMA and ionomycin stimulation reduced the Foxp3 expression levels (lower left panel, Figure 2), the expression of all 3 cytokines in CD4^+^CD25^+^CCR4^+^ T cells was significantly decreased (Figures 4A, B).

Results of the intracellular flow cytometry revealed that CD4^+^CD25^+^CCR4^+^Foxp3^−^ T cells exhibited increased IFN-γ expression and decreased IL-2 and IL-17 expression in HAM/TSP. Therefore, we wished to determine whether comparable changes in the mRNA expression of cytokines could also be demonstrated using PBMCs before culture. Therefore, CD4^+^CD25^+^CCR4^+^ T cells from PBMCs of 3 HDs, 4 HAM/TSP patients, and 3 ATL patients were isolated by FACS sorting, and the mRNA expression levels of IFN-γ, IL-2, and IL-17 were quantified by real-time RT-PCR (Figure 4C). These results were in agreement with those of the cytokine protein expression obtained by flow cytometry (Figures 4A, B). The mRNA expression of IFN-γ in CD4^+^CD25^+^CCR4^+^ T cells was increased in HAM/TSP patients than in the HDs (Figure 4C). Similarly, IL-2 and IL-17 mRNA levels in CD4^+^CD25^+^CCR4^+^ T cells were decreased in HAM/TSP patients than in the HDs (Figures 4A, B). Collectively, these results indicate that the differential production of proinflammatory cytokines in CD4^+^CD25^+^CCR4^+^ cells is dependent on the Foxp3 expression level. In HAM/TSP patients, HTLV-1-infected CD4^+^CD25^+^CCR4^+^Foxp3^−^ T cells overproduce IFN-γ and produce low levels of IL-17.

### CD4^+^CD25^+^CCR4^+^ T cells in HAM/TSP produce low levels of IL-4 and IL-10

**Figure 5:**
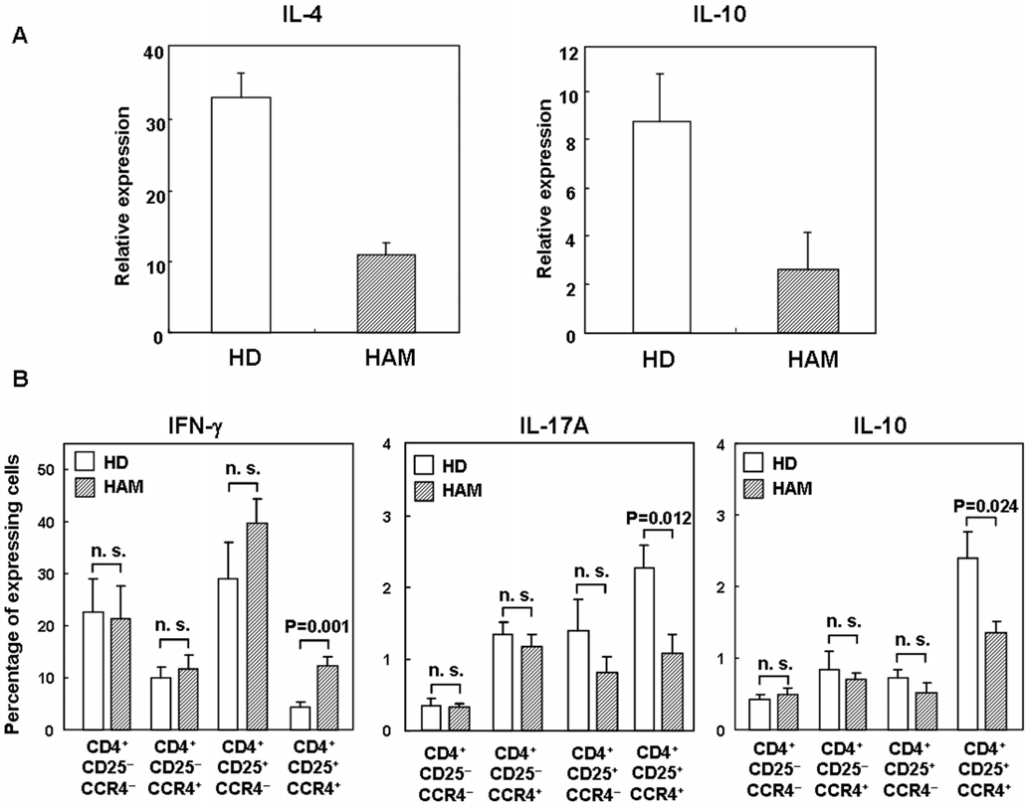
CD4^+^CD25^+^CCR4^+^ T cells in HAM/TSP produce lower levels of IL-4 and IL-10. A. Decreased mRNA expression levels of IL-4 and IL-10 in CD4^+^CD25^+^CCR4^+^ T cells from HAM/TSP patients. CD4^+^CD25^+^CCR4^+^ T cells were separated by FACS sorting from 4 HDs and 4 HAM/TSP patients, and the IL-4 or IL-10 mRNA expression levels were analyzed by real-time RT-PCR. Data are presented as mean±SE. B. The percentage of cytokine (IFN-γ, IL-10, and IL-17A)-expressing cells in each cell subtype between HDs and HAM/TSP patients. CD4^+^ T cells were separated using magnetic beads from PBMC of 8 HDs and 7 HAM/TSP patients, and stimulated by PMA and ionomycin for 5 hours in the presence of monensin. Then, the percentage of IFN-γ-, IL-10-, and IL-17-positive cells in each cell population was determined by FACS. Data are presented as mean±SE. The P value was calculated by unpaired Student's *t* test. n.s. not significant.

Although the overproduction of IFN-γ in CD4^+^CD25^+^CCR4^+^ T cells of HAM/TSP patients was demonstrated (Figure 4), it was also of interest to determine whether the production of suppressive cytokines such as IL-4 and IL-10 was decreased in these cells because the CD4^+^CD25^+^CCR4^+^ T cells in healthy donors mainly include Th2 and Treg cells [26]. This T cell subset was separated from PBMCs by FACS sorting from 4 HDs and 4 HAM/TSP patients, and the IL-4 or IL-10 mRNA expression levels were analyzed by real-time RT-PCR. As shown in Figure 5A, CD4^+^CD25^+^CCR4^+^ T cells in HAM/TSP produced lower levels of IL-4 and IL-10 than in HDs.

Furthermore, to examine whether these functional alterations in the CD4^+^CD25^+^CCR4^+^ T cells from HAM/TSP patients are specific to the cell subtype, we investigated the intracellular cytokine expression of IFN-γ, IL-17, and IL-10 in each of the 4 cell subtypes from 8 HDs and 7 HAM/TSP patients by FACS after PMA and ionomycin stimulation (Figure 5B). In CD4^+^CD25^+^CCR4^+^ T cells, the percentage of IFN-γ-producing cells was low and that of IL-10- and IL-17-producing cells was high in HDs, whereas the percentage of IFN-γ-producing cells was increased and that of IL-10- and IL-17-producing cells was decreased in HAM/TSP patients, confirming the results presented in Figure 4 and Figure 5A. Importantly, these alterations were not observed in other cell subtypes (Figure 5B), and this functional alteration in cytokine production was specific to the CD4^+^CD25^+^CCR4^+^ T cells of HAM/TSP patients.

### Increased HTLV-1 *tax* expression in CD4^+^CD25^+^CCR4^+^ cells of HAM/TSP patients

**Figure 6:**
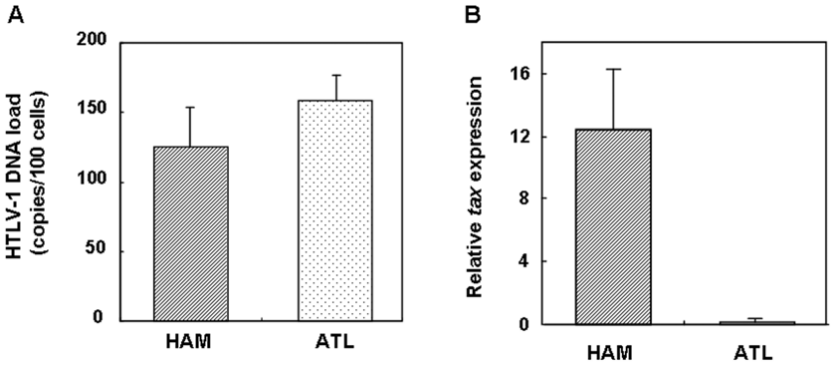
Increased HTLV-1 *tax* expression in CD4^+^CD25^+^CCR4^+^ cells from HAM/TSP patients. HTLV-1 proviral DNA load and HTLV-1 *tax* expression in CD4^+^CD25^+^CCR4^+^ cells from HAM/TSP and ATL patients. The HTLV-1 proviral DNA load was measured in the CD4^+^CD25^+^CCR4^+^ cells from HAM/TSP and ATL patients (left panel, n = 3), and the HTLV-1 *tax* mRNA expression (right panel, HAM: n = 4, ATL: n = 3) was quantitatively measured. Data are presented as mean±SE.

Our results demonstrated differential proinflammatory cytokine production in CD4^+^CD25^+^CCR4^+^ cells between HAM/TSP and ATL that is dependent on the level of Foxp3 expression (Figure 4). Since we have previously demonstrated that HTLV-1 *tax* can reduce Foxp3 expression [20], it is possible that the intracellular expression of HTLV-1 may function to direct T cell differentiation from Treg cells (Foxp3^+^ and no cytokine production) into non-Treg cells (Foxp3^−^ with inflammatory cytokine production). Therefore, we analyzed the differences in the HTLV-1 DNA load and the HTLV-1 *tax* expression of CD4^+^CD25^+^CCR4^+^ T cells between HAM/TSP and ATL patients. We isolated the CD4^+^CD25^+^CCR4^+^ T cells from the PBMCs of HAM/TSP and ATL patients and quantified the HTLV-1 provirus DNA load and HTLV-1 *tax* mRNA expression by real-time PCR and RT-PCR, respectively. As shown in Figure 6, while the HTLV-1 provirus DNA loads between the 2 groups were equivalent, the HTLV-1 *tax* mRNA expression in CD4^+^CD25^+^CCR4^+^ T cells was significantly increased in the HAM/TSP patients than in the ATL patients.

### The frequency of IFN-γ producing CD4^+^CD25^+^CCR4^+^ T cells correlates with disease severity in HAM/TSP

**Figure 7:**
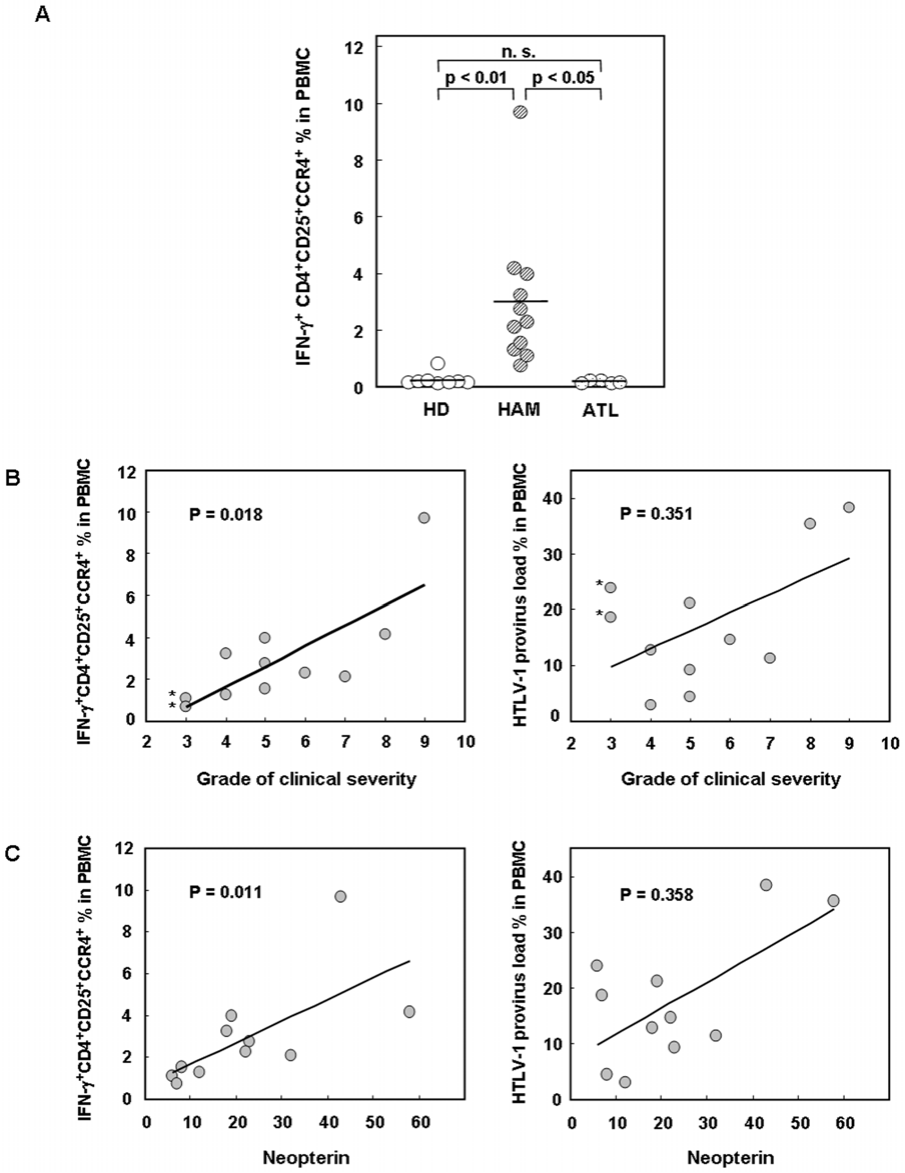
Frequency of IFN-γ-producing CD4^+^CD25^+^CCR4^+^Foxp3^−^ T cells is increased and correlates with HAM/TSP disease severity. A. Percentage of IFN-γ^+^CD4^+^CD25^+^CCR4^+^ T cells to the total PBMCs in 8 HDs, 11 HAM/TSP patients, and 5 ATL patients. The horizontal bar indicates the mean value of each group. Scheffe's test (nonparametric) was used for statistical analysis. n.s. not significant. B. Correlation between HAM/TSP disease severity and the frequency of IFN-γ-producing CD4^+^CD25^+^CCR4^+^ T cells in PBMCs or the HTLV-1 provirus load in PBMCs. According to Spearman's rank correlation test, the frequency of IFN-γ^+^CD4^+^CD25^+^CCR4^+^ T cells correlated with disease severity (p = 0.018), while the HTLV-1 provirus load did not (p = 0.351). * indicates HAM/TSP patients with disease severity of a low grade and high provirus load. C. Correlation between neopterin concentration in CSF and the frequency of IFN-γ-producing CD4^+^CD25^+^CCR4^+^ T cells in PBMCs or the HTLV-1 provirus load in PBMCs. According to Spearman's rank correlation test, the frequency of IFN-γ^+^CD4^+^CD25^+^CCR4^+^ T cells correlated with CSF neopterin concentration (p = 0.011), while the HTLV-1 provirus load did not (p = 0.358).

In this study, we demonstrated that IFN-γ production is specifically increased in the CD4^+^CD25^+^CCR4^+^ T cells in HAM/TSP patients (Figures 4 and 5). We then measured the percentages of the IFN-γ-producing CD4^+^CD25^+^CCR4^+^ T cells to the total PBMCs in 8 HDs, 11 HAM/TSP, and 5 ATL patients. As shown in Figure 7A, the frequency of this population was significantly and specifically increased in HAM/TSP patients than in HDs (p<0.01) and ATL patients (p<0.05).

In order to determine a functional consequence of this population in the pathogenesis of HAM/TSP, we aimed to analyze whether the frequency of IFN-γ-producing CD4^+^CD25^+^CCR4^+^ T cells was correlated with disease activity. Therefore, we studied the correlation between the frequency of IFN-γ-producing CD4^+^CD25^+^CCR4^+^ T cells with a HAM/TSP clinical severity score and the concentration of neopterin in CSF. Neopterin is known to be a marker of active inflammation in the spinal cord lesions of HAM/TSP patients [33], [34]. The frequency of IFN-γ-producing CD4^+^CD25^+^CCR4^+^ T cells correlated with disease severity (Figure 7B, p = 0.018) and CSF neopterin concentrations (Figure 7C, p = 0.011), while the HTLV-1 provirus DNA load in PBMCs did not correlate with disease severity and CSF neopterin concentrations (p = 0.351 and p = 0.358, respectively). It is notable that in the patients with low grade disease severity and high virus load (indicated by * in Figure 7B), the IFN-γ production in HTLV-1-infected T cells (CD4^+^CD25^+^CCR4^+^) was low. These results suggest that the number of functionally altered (IFN-γ producing) HTLV-1-infected T cells rather than the absolute number of HTLV-1-infected T cells is more important as a correlating factor for HAM/TSP disease activity and prognosis.

### Removal of CD4^+^CD25^+^CCR4^+^ T cells decreases spontaneous proliferation of PBMCs from HAM/TSP patients

**Figure 8:**
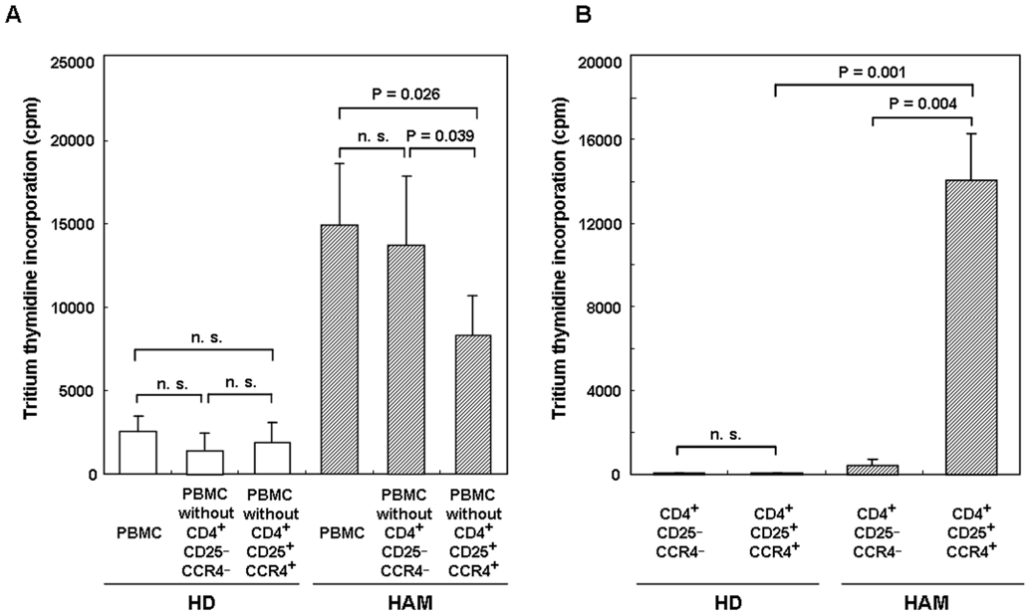
Removal of CD4^+^CD25^+^CCR4^+^ T cells decreases spontaneous proliferation of PBMCs from HAM/TSP patients. A. PBMCs, PBMC lacking CD4^+^CD25^+^CCR4^+^, and PBMCs lacking CD4^+^CD25^−^CCR4^−^ obtained using FACS sorting from 4 HDs and 4 HAM/TSP patients were cultured for 7 days, and the magnitude of proliferation was compared. Data are presented as mean±SE. The P values are calculated by paired *t* test. n.s. not significant. B. CD4^+^CD25^+^CCR4^+^ T cells of HAM/TSP patients proliferated independently. CD4^+^CD25^+^CCR4^+^ cells and CD4^+^CD25^−^CCR4^−^ T cells were separated by FACS sorting from 4 HDs and 4 HAM/TSP patients, cultured for 7 days, and the magnitude of proliferation was compared. Data are presented as mean±SE. The P values within each group are calculated by paired *t* test and those between the HD and HAM groups, by unpaired Student's *t* test. n.s. not significant.

Although Th2 and Treg CD4^+^CD25^+^CCR4^+^ T cells in healthy individuals are known to be immunosuppressive [26], we demonstrated that in HAM/TSP patients, HTLV-1-infected CD4^+^CD25^+^CCR4^+^ T cells overproduce Th1 cytokines such as IFN-γ and produce low levels of suppressive cytokines such as IL-4 and IL-10. Therefore, we determined if the CD4^+^CD25^+^CCR4^+^ T cells in HAM/TSP patients were also functionally immunosuppressive. Because the HAM/TSP PBMCs are known to proliferate spontaneously without any mitogenic stimuli [35], PBMCs, PBMCs lacking CD4^+^CD25^+^CCR4^+^ T cells, and PBMCs lacking CD4^+^CD25^−^CCR4^−^ T cells were prepared using FACS sorting from 4 HDs and 4 HAM/TSP patients and cultured for 7 days, after which the magnitude of proliferation between the 2 groups was compared. As shown in Figure 8A, spontaneous proliferation of HAM PBMCs was significantly reduced in the PBMCs lacking CD4^+^CD25^+^CCR4^+^ T cells (p = 0.026) but not in the PBMCs lacking CD4^+^CD25^−^CCR4^−^ T cells. In healthy donors, no difference in spontaneous proliferation was observed between PBMCs lacking CD4^+^CD25^+^CCR4^+^ T cells and those lacking CD4^+^CD25^−^CCR4^−^ T cells (Figure 8A). This result suggests that CD4^+^CD25^+^CCR4^+^ T cells are a necessary component of the observed spontaneous lymphoproliferative response in the PBMCs of HAM/TSP patients.

We then examined the proliferation ability of CD4^+^CD25^+^CCR4^+^ T cells in HAM/TSP patients. We separated CD4^+^CD25^+^CCR4^+^ T cells and CD4^+^CD25^−^CCR4^−^ T cells from 4 HDs and 4 HAM/TSP patients by FACS sorting and cultured them for 7 days, following which we compared the magnitude of proliferation between these cell populations (Figure 8B). CD4^+^CD25^+^CCR4^+^ T cells from HAM/TSP patients proliferated independently, while CD4^+^CD25^−^CCR4^−^ T cells from HAM/TSP patients and CD4^+^CD25^+^CCR4^+^ and CD4^+^CD25^−^CCR4^−^ T cells from HDs did not.

## Discussion

HAM/TSP is a neuroinflammatory disorder etiologically associated with a human retrovirus (HTLV-1). The pathogenesis of this disorder has been demonstrated to involve HTLV-1-infected T cells, which induce chronic hyperimmune responses that can lead to an immunopathological process in the CNS of genetically susceptible infected individuals [11], [36]. We hypothesized that there may exist subpopulations of chronically activated “pathogenic T cells” in HTLV-1-infected cells of HAM/TSP patients that may function to accelerate inflammation and that these cells could be an important target for clinical therapeutic interventions. It was important to initially determine markers of HTLV-1-infected T cells in HAM/TSP patients. In our previous studies, we have demonstrated that CD4^+^CD25^+^ T cells are the predominant viral reservoir in the peripheral blood of HAM/TSP patients [12], although CD8^+^ T cells [14], macrophages [13], [16] and dendritic cells [15] are also infected in HAM/TSP. Recently, the chemokine receptor CCR4 has been shown to be expressed on HTLV-1-infected ATL cells [22]. Since CCR4 is known to be selectively expressed on Th2, Th17, and Treg cells [23], [26], and since ATL cells have been shown to express high levels of Foxp3, a specific marker of Treg [22], [31], it was hypothesized that ATL cells may be derived from Treg [37]. However, in HAM/TSP patients, we and other researchers have demonstrated that CD4^+^CD25^+^ T cells exhibit reduced Foxp3 expression and Treg suppression [20], [27]–[29]. Furthermore, it has been demonstrated that HTLV-1-infected CD4^+^ T cells in HAM/TSP patients produce Th1 cytokines (IFN-γ) [20], [32]. Therefore, it was expected that CD4^+^CD25^+^ T cells might exhibit reduced CCR4 expression in HAM/TSP patients. However, we clearly demonstrated that CCR4 was selectively overexpressed on HTLV-1-infected T cells. We also demonstrated that the majority of CD4^+^CD25^+^CCR4^+^ T cells were infected with HTLV-1 and that this T cell subset was increased in HAM/TSP patients (Figure 1). Furthermore, CD4^+^CD25^+^CCR4^+^ T cells, which mainly include suppressive T cell subsets such as Th2 and Treg in HTLV-1-seronegative healthy controls [26], become Th1-like cells with overproduction of IFN-γ and production of low levels of IL-4 and IL-10 (Figures 4 and 5) in patients with HAM/TSP. It is noteworthy that this functional alteration in cytokine production was specific to the HTLV-1-infected CD4^+^CD25^+^CCR4^+^ T cell subtype (Figure 5B). These results suggest that HTLV-1-infected CD4^+^CD25^+^CCR4^+^ T cells in HAM/TSP patients were functionally proinflammatory rather than suppressive. A hallmark of hyperimmune responsiveness in HAM/TSP is the capacity of PBMCs to spontaneously proliferate, and the removal of CD4^+^CD25^+^CCR4^+^ T cells diminished this proliferative response (Figure 8A). Recently, Shimizu et al reported that the depletion of CCR4^+^ cells reduced IFN-γ production in the supernatant of cultured antigen-responding cells in HAM/TSP patients [44]. Thus, functionally altered CD4^+^CD25^+^CCR4^+^ T cells in HAM/TSP patients are a necessary component of the accelerated lymphoproliferative response.

Patients with HAM/TSP exhibit a chronic inflammatory disorder characterized by accumulation of activated CD4^+^ and CD8^+^ T cells [38]. CD4^+^CD25^+^ T cells from HAM/TSP patients also exhibit an activated phenotype marked by decreased expression of CD45RA together with increased expression of IFN-γ compared to the same subset in healthy individuals [20]. Furthermore, we previously demonstrated that HTLV-1 *tax* overexpression was capable of reducing Foxp3 expression, and the HTLV-1 *tax* mRNA expression in PBMCs of HAM/TSP was elevated [8], [20]. Therefore, we hypothesized that persistent T cell activation in patients with HAM/TSP triggered by viral antigens such as HTLV-1 *tax* together with the downregulation of Foxp3 may result in decline of the CD4^+^CD25^+^CCR4^+^Foxp3^+^ Treg population and accumulation of Foxp3-negative CD4^+^CD25^+^CCR4^+^ T cells that lack suppressive function, but are capable of exacerbating the disease process. In this study, we have demonstrated that CD4^+^CD25^+^CCR4^+^Foxp3^−^ T cells were proliferating *in vivo* (Ki67 positive) (Figure 3), and the frequency of this population was increased in HAM/TSP patients (Figure 2). Moreover, analysis of the cytokine expression in this CD4^+^CD25^+^CCR4^+^Foxp3^−^ T cell subset demonstrated that these cells were unique because they produce multiple proinflammatory cytokines such as IL-2, IL-17, and few IFN-γ in healthy individuals while CD4^+^CD25^+^CCR4^+^Foxp3^+^ T cells (Treg cells) did not (Figure 4). Furthermore, it was demonstrated that HAM/TSP patients exhibited only few CD4^+^CD25^+^CCR4^+^Foxp3^+^ T cells that do not produce such cytokines (Figure 3). Rather, the CD4^+^CD25^+^CCR4^+^Foxp3^−^ T cells in HAM/TSP were increased and found to overproduce IFN-γ (Figures 3 and 4). Further, the frequency of these IFN-γ-producing CD4^+^CD25^+^CCR4^+^Foxp3^−^ T cells may have a functional consequence since this population was associated with increased clinical disease activity and severity in HAM/TSP (Figure 7). These results suggest that IFN-γ-producing CD4^+^CD25^+^CCR4^+^Foxp3^−^ T cells may play an important pathogenic role in HAM/TSP by augmenting inflammation in the CNS. Thus, we have defined a unique T cell subset—IFN-γ^+^CCR4^+^Foxp3^−^CD4^+^CD25^+^ T cells—that is specifically increased in HAM/TSP (tentatively designated T_HAM_ cells) and is rarely encountered in healthy individuals. Since some HAM/TSP patients are known to experience complications with other autoimmune disorders [5], [6], it would be of interest to determine if this newly defined T cell subset (T_HAM_ cells: IFN-γ^+^CCR4^+^Foxp3^−^CD4^+^CD25^+^ T cells) may also be abnormally increased and functionally deregulated in other immunological diseases.

Although CD4^+^CD25^+^CCR4^+^ T cells are predominantly infected by HTLV-1 in both HAM/TSP and ATL (Figure 6), it was demonstrated that the ratio of T_HAM_ cells (CCR4^+^Foxp3^−^ with IFN-γ production) to Treg cells (CCR4^+^Foxp3^+^ with no cytokine production) in the CD4^+^CD25^+^CCR4^+^ T cell subset were high in HAM/TSP and low in ATL (Figure 2). This differential T_HAM_/Treg ratio in HTLV-1-infected T cells may be associated with the different immune responses observed between HAM/TSP and ATL. ATL patients have very low frequencies of Tax-specific CD8^+^ T cells in PBMCs and tend to develop opportunistic infections [21], while HAM/TSP is characterized by extraordinarily high levels of Tax-specific CD8^+^ CTL [7], [8], [11], [40], [41]. It has been reported that the immunosuppressive function of CD4^+^CD25^+^ T cells with high expression of Foxp3 in ATL patients is intact [42]. Thus, the CD4^+^CD25^+^CCR4^+^ leukemia T cells with increased Treg function may also contribute to the clinically observed cellular immunodeficiency in ATL patients. However, HAM/TSP patients show extremely high cellular and humoral immune responses such as high frequencies of Tax-specific CD8^+^ T cells as well as cytomegalovirus (CMV)-specific CD8^+^ T cells in PBMCs [7], [8], [28]; high antibody titer to HTLV-1 [6]; and increased production of proinflammatory cytokines such as IL-6, IL-12, and IFN-γ [9]. It has been reported that HAM CD4^+^CD25^+^ T cells with low expression of Foxp3 [20] or HTLV-1 Tax-expressing Foxp3^+^ Treg cells [45] are defective in their immunosuppressive function. Here, we demonstrate that HTLV-1-infected IFN-γ overproducing CD4^+^CD25^+^CCR4^+^Foxp3^−^ T cells (T_HAM_ cells) are increased in HAM/TSP patients, and these levels can be correlated with disease severity. Thus, CD4^+^CD25^+^CCR4^+^ T cells with increased proinflammatory function together with a defective Treg compartment [20], [27]–[29] may overcome the regulatory effect of HTLV-1-uninfected Treg cells [45] and at least partly account for the heightened immune response observed in HAM/TSP patients. Collectively, these observations support the hypothesis that imbalance of the T_HAM_/Treg ratio in HTLV-1-infected CD4^+^CD25^+^CCR4^+^ T cells is an important factor that contributes to immunological differences of the host immune response between HAM/TSP and ATL (Figure S2).

The high Foxp3 expression without cytokine production and low Foxp3 expression with cytokine production in CD4^+^CD25^+^CCR4^+^ T cells (Figure 3A) are consistent with recent reports demonstrating that Foxp3 can target and repress the transcriptional activity of NFAT-AP-1 complexes present at the cytokine promoter [43]. We have previously reported that overexpression of HTLV-1 *tax* was capable of reducing Foxp3 expression [20]. As we have demonstrated high HTLV-1 *tax* expression in HAM/TSP CD4^+^CD25^+^CCR4^+^ T cells (Foxp3^−^) and low HTLV-1 *tax* expression in ATL CD4^+^CD25^+^CCR4^+^ T cells (Foxp3^+^) (Figure 6), it was suggested that HTLV-1 expression intracellularly may act as a “switch” that directs T cell differentiation of CD4^+^CD25^+^CCR4^+^ T cells from Foxp3^+^ to IFN-γ^+^Foxp3^−^ T cells. Further, the increased IFN-γ production and decreased IL-17 production in CD4^+^CD25^+^CCR4^+^Foxp3^−^ T cells of HAM/TSP patients (Figures 4 and 5) suggests that HTLV-1 expression may also contribute to the differentiation of Th1 versus Th17 CD4^+^ T cells. These hypotheses are currently under investigation to elucidate the precise molecular mechanisms by which HTLV-1 influences the fate and function of CD4^+^CD25^+^CCR4^+^ T cells.

In conclusion, we have defined a unique T cell population (T_HAM_ cells: IFN-γ^+^CCR4^+^Foxp3^−^CD4^+^CD25^+^ T cells) that are rarely encountered in normal individuals, are infected by HTLV-1, and are found to be abnormally increased and proinflammatory and correlate with disease severity in HAM/TSP patients. This study is the first to demonstrate that T_HAM_ cells are crucial for the pathogenesis of this retrovirus-associated chronic inflammatory disorder of the nervous system.

## Materials and Methods

### Subjects, cell preparation, and determination of CSF neopterin concentrations

A total of 11 HAM/TSP patients, 8 healthy donors, and 5 ATL patients (chronic type) participated in this study. Written informed consents were obtained from all the subjects in accordance with the Declaration of Helsinki as part of a clinical protocol reviewed and approved by the Institutional Ethics Committee (St. Marianna University). Blood samples were collected from the subjects, peripheral blood mononuclear cells (PBMCs) were separated by centrifugation over Ficoll-Hypaque gradients, and the cells were cryopreserved in liquid nitrogen until testing. HAM/TSP was diagnosed according to the guidelines defined by the WHO, and ATL was diagnosed on the basis of the criteria proposed by Shimoyama [46]. HTLV-1 seropositivity was determined by particle agglutination (Serodia-HTLV-1; Fujirebio, Japan) and confirmed by western blotting (SRL, Japan). Clinical disease severity was evaluated by Osame's motor disability scale (Table 1) [6]. In some experiments, CD4^+^ T cells were negatively selected from the PBMCs by using magnetic beads (MACS CD4^+^ T cell isolation kit; Miltenyi Biotec, Germany), according to the manufacturer's instructions. FACS cell sorting was performed using JSAN (Bay Bioscience, Japan), and purity after sorting was approximately 99% (Figure S1). Neopterin concentration was measured by high-performance liquid chromatography with fluorometric detection methods [33] (SRL, Japan).

**Table 1:** Motor disability grading for HAM/TSP.

Grade	Motor disability
0	Normal gait and running
1	Normal gait but runs slowly
2	Abnormal gait (staggering or spastic)
3	Abnormal gait and unable to run
4	Needs support while using stairs but walks without assistance
5	Needs one hand support in walking
6	Needs two hands support in walking (can walk more than 10 meter)
7	Needs two hands support in walking (can walk less than 10 meter)
8	Needs two hands support in walking (can walk less than 5 meter)
9	Unable to walk but can walk on all fours
10	Unable to walk on all fours but can crawl with hands
11	Unable to crawl with hands but can turn sideways in bed
12	Unable to turn sideways but can move the toes
13	Completely bedridden (unable to move the toes)

### Flow cytometry

Cells were immunostained with various combinations of the following fluorescence-conjugated antibodies that served as cell surface markers: CD4 (OKT4; eBioscience, San Diego, CA), CD25 (M-A251; BD Biosciences, San Diego, CA), CCR4 (1G1; BD Biosciences). In some experiments, cells were fixed by a staining buffer set (eBioscience), then intracellularly stained with the antibodies to Foxp3 (PCH101; eBioscience) and Ki67 (B56; BD Biosciences). For intracellular cytokine staining, the cells were stimulated for 5 h with phorbol 12-myristate 13-acetate (PMA) and ionomycin (Sigma, Japan) in the presence of monensin (Golgistop; BD Biosciences). Cells were fixed and stained with the antibodies to IFN-γ (B27; BD Biosciences), IL-2 (MQ1-17H12; BD Biosciences), IL-10 (JES3-9D7, eBioscience), and IL-17A (eBio64DEC17; eBioscience). Flow cytometric analysis was performed on a FACSCalibur cytometer (BD Biosciences). Data processing was performed using FlowJo software (TreeStar, San Diego, CA).

### Real-time PCR

The HTLV-1 proviral DNA load was measured as previously described [8]. For the real-time reverse transcriptase-PCR (RT-PCR) analysis, CD4^+^CD25^+^CCR4^+^ cells were separated by FACS sorting from 3 HDs, 4 HAM/TSP patients, and 3 ATL patients. The number of subjects for RT-PCR was limited because a large number of PBMCs are required for sorting a sufficient number of CD4^+^CD25^+^CCR4^+^ cells for the analysis. Total RNA was isolated from cells using ISOGENE (Nippon Gene, Japan). The first-strand cDNA was synthesized with random hexamers and reverse transcriptase (ReverTraAce; Toyobo, Japan) using 1 µg of total RNA in a reaction volume of 20 µl. Real-time PCR reactions were carried out using TaqMan Universal Master Mix (Applied Biosystems) and Universal Probe Library assays designed using ProbeFinder software (Roche Applied Science). The primer sequences and Universal Probe numbers used are available upon request (Roche Applied Science). ABI Prism 7500 SDS was programmed to an initial step of 2 min at 50°C and 10 min at 95°C, followed by 45 cycles of 15 sec at 95°C and 1 min at 60°C. The primers used were as follows: IFN-γ, 5′-GGCATTTTGAAGAATTGGAAAG-3′ (forward) and 5′-TTTGGATGCTCTGGTCATCTT-3′ (reverse) (probe No. 21); IL-2, 5′-AAGTTTTACATGCCCAAGAAGG-3′ (forward) and 5′-AAGTGAAAGTTTTTGCTTTGAGCTA-3′ (reverse) (probe no. 65); IL-17A, 5′-TGGGAAGACCTCATTGGTGT-3′ (forward) and 5′-GGATTTTCGTGGGATTGTGAT-3′ (reverse) (probe No. 8); and GAPDH, 5′-AGCCACATCGCTCAGACA-3′ (forward) and 5′-GCCCAATACGACCAAATCC-3′ (reverse) (probe no. 60). The primers and probe for detecting the HTLV-1 *tax* mRNA load were used as described previously [8]. The primers and probe for IL-4 and IL-10 were used from TaqMan® Gene Expression Assays (IL-4: Hs: Hs00932431_m1, IL-10: Hs00174086_m1). Relative quantification of mRNA was performed using the comparative threshold cycle method using GAPDH as an endogenous control. For each sample, target gene expression was normalized to the expression of GAPDH. To determine the relative expression levels, the following formula was used: target gene expression = 2^−(Ct[target] − Ct[GAPDH])^.

### Proliferation assays

Separated PBMCs, PBMCs lacking CD4^+^CD25^+^CCR4^+^ cells, PBMCs lacking CD4^+^CD25^−^CCR4^−^ cells, CD4^+^CD25^+^CCR4^+^ T cells, and CD4^+^CD25^−^CCR4^−^ T cells from HAM/TSP patients were plated into 96-well round bottom plates (1×10^5^ per well) without any mitogenic stimuli. RPMI 1640 with l-Glutamine (Wako, Japan) supplemented with 5% human AB serum (Gibco-Invitrogen, NY), penicillin, and streptomycin (Wako, Japan) was used as the culture medium. After 6 days of culture, 1 µCi tritium thymidine was added to each well, and the cells were cultured for an additional 16 hours. A liquid scintillation counter was used to measure the proliferation. Cultures were performed in triplicate for each experiment, and the average proliferation data was used for analysis.

### Statistical analyses

The Wilcoxon signed-rank test was used to compare the HTLV-1 provirus load between CD4^+^CD25^+^CCR4^+^ and CD4^+^CD25^+^CCR4^−^ cells. An unpaired Student's *t* test was used to compare the frequency of CD4^+^CD25^+^CCR4^+^Foxp3^−^ T cells in PBMCs, to compare the percentage of the Foxp3^+^ population in CD4^+^CD25^+^CCR4^+^ T cells, to compare the frequency of CD4^+^CD25^−^CCR4^−^, CD4^+^CD25^−^CCR4^+^, CD4^+^CD25^+^CCR4^−^ or CD4^+^CD25^+^CCR4^+^ T cells, to compare the percentage of IFN-γ, IL-10 or IL-17 positive cells in each cell population, and to compare the magnitude of proliferation of CD4^+^CD25^+^CCR4^+^ T cells between HDs and HAM/TSP patients. Paired Student's *t* test was used to compare the proliferative response of HAM PBMCs with and without CD4^+^CD25^+^CCR4^+^ T cells and HAM PBMCs with and without CD4^+^CD25^−^CCR4^−^ T cells, and to compare the proliferative response between CD4^+^CD25^+^CCR4^+^ T cells and CD4^+^CD25^−^CCR4^−^ T cells within HDs or HAM/TSP patients. The nonparametric Scheffe's test for multiple comparison was used to compare the data between HAM/TSP patients, ATL patients, and HDs. Spearman's rank correlation test was used to investigate the correlation between the percentage of IFN-γ-producing CD4^+^CD25^+^CCR4^+^ T cells in PBMCs, HTLV-1 *tax* proviral load in PBMCs, clinical severity score, and concentration of neopterin in the CSF. Statistical analyses were performed using Statcel2 software.

## Supporting Information

Figure S1 Purity of cell populations separated by FACS sorting. CD4+CD25−CCR4−, CD4+CD25−CCR4+, CD4+CD25+CCR4−, and CD4+CD25+CCR4+T cells were separated by FACS. The purity of each population was indicated as the histogram of fractions from (a)–(d): (a) CD4+CD25+CCR4−, (b) CD4+CD25+CCR4+, (c) CD4+CD25−CCR4−, and (d) CD4+CD25−CCR4+T cells. The purity of each population is approximately 99%.
(0.60 MB TIF)

Figure S2 Schematic hypothesis outlining the importance of the different characteristics of HTLV-1-infected T cells in HAM/TSP and ATL patients. An imbalance in the THAM/Treg ratio in HTLV-1-infected CD4+CD25+CCR4+T cells may be an important factor that contributes to immunological differences of the host immune response between HAM/TSP and ATL.
(0.34 MB TIF)
