# Supplementary figures and images for: Abnormally High Levels of Virus-Infected IFN-γ+CCR4+CD4+CD25+ T Cells in a Retrovirus-Associated Neuroinflammatory Disorder
Source: PLoS One. 2009 Aug 5;4(8):e6517. doi: 10.1371/journal.pone.0006517 (PMC2715877; doi:10.1371/journal.pone.0006517)

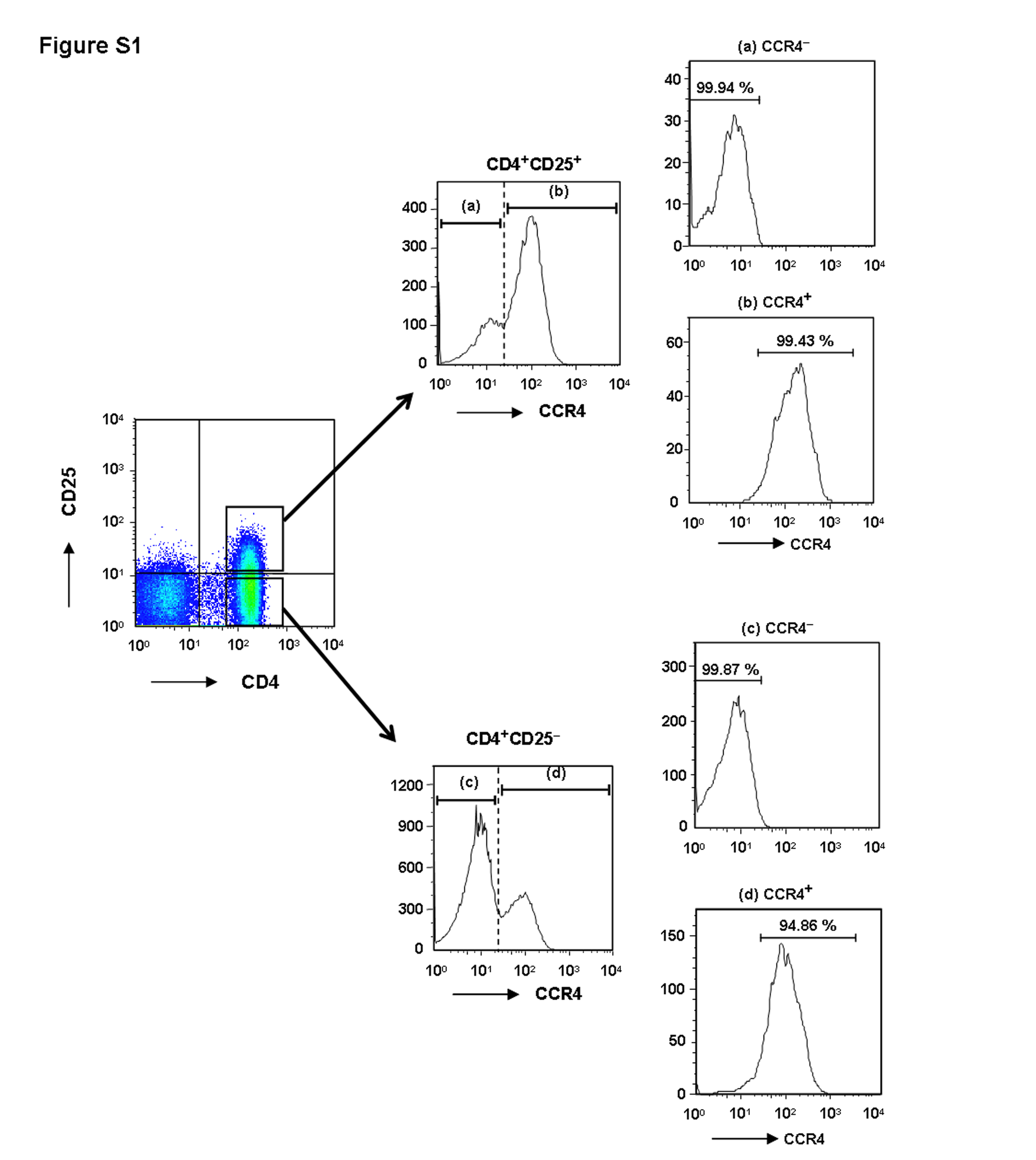

Supplement: Figure S1 — Purity of cell populations separated by FACS sorting. CD4+CD25−CCR4−, CD4+CD25−CCR4+, CD4+CD25+CCR4−, and CD4+CD25+CCR4+T cells were separated by FACS. The purity of each population was indicated as the histogram of fractions from (a)–(d): (a) CD4+CD25+CCR4−, (b) CD4+CD25+CCR4+, (c) CD4+CD25−CCR4−, and (d) CD4+CD25−CCR4+T cells. The purity of each population is approximately 99%. (0.60 MB TIF) [file pone.0006517.s001.tif]

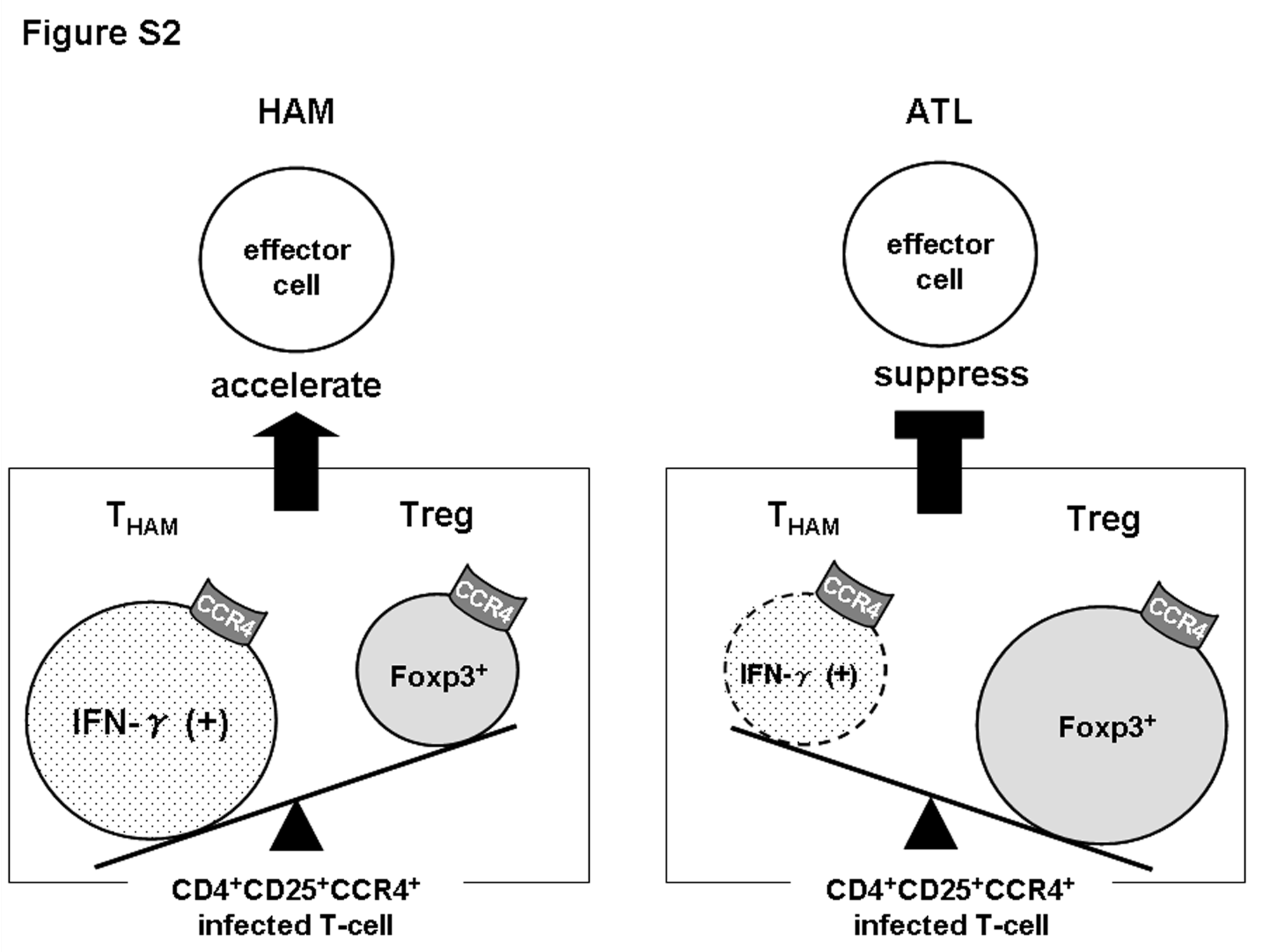

Supplement: Figure S2 — Schematic hypothesis outlining the importance of the different characteristics of HTLV-1-infected T cells in HAM/TSP and ATL patients. An imbalance in the THAM/Treg ratio in HTLV-1-infected CD4+CD25+CCR4+T cells may be an important factor that contributes to immunological differences of the host immune response between HAM/TSP and ATL. (0.34 MB TIF) [file pone.0006517.s002.tif]
